# Cytokine Alterations in Schizophrenia: An Updated Review

**DOI:** 10.3389/fpsyt.2019.00892

**Published:** 2019-12-06

**Authors:** Sara Momtazmanesh, Ameneh Zare-Shahabadi, Nima Rezaei

**Affiliations:** ^1^School of Medicine, Tehran University of Medical Sciences, Tehran, Iran; ^2^Neuroimmunology Research Association (NIRA), Universal Scientific Education and Research Network (USERN), Tehran, Iran; ^3^Department of Molecular Biology, University of Louisiana at Lafayette, Lafayette, LA, United States; ^4^Research Center for Immunodeficiencies, Pediatrics Center of Excellence, Children’s Medical Center, Tehran University of Medical Sciences, Tehran, Iran; ^5^Department of Immunology, School of Medicine, Tehran University of Medical Sciences, Tehran, Iran; ^6^Network of Immunity in Infection, Malignancy and Autoimmunity (NIIMA), Universal Scientific Education and Research Network (USERN), Tehran, Iran

**Keywords:** schizophrenia, cytokines, inflammation, behavioral symptoms, treatment outcome, antipsychotic agents

## Abstract

Schizophrenia, a multisystem disorder with an unknown etiology, is associated with several immune dysfunctions, including abnormal levels of circulating cytokines. In this review, we investigated the changes of cytokines in schizophrenic patients, their connection with behavioral symptoms severity and their potential clinical implications. We also assessed the possible causative role of abnormal cytokine levels in schizophrenia pathogenesis. Based on meta-analyses, we categorized cytokines according to their changes in schizophrenic patients into four groups: (1) increased cytokines, including interleukin (IL)-6, tumor necrosis factor (TNF)-α, IL-1β, IL-12, and transforming growth factor (TGF)-β, (2) non-altered cytokines, including IL-2, IL-4, and IL-17, (3) increased or non-altered cytokines, including IL-8 and interferon (IFN)-γ, and (4) IL-10 with increased, decreased, and non-altered levels. Notably, alterations in cytokines may be variable in four different categories of SP, including first-episode and drug-naïve, first-episode and non-drug-naïve, stable chronic, and chronic in acute relapse. Furthermore, disease duration, symptoms severity, incidence of aggression, and cognitive abilities are correlated with levels of certain cytokines. Clinical implications of investigating the levels of cytokine in schizophrenic patients include early diagnosis, novel therapeutic targets development, patient stratification for choosing the best therapeutic protocol, and predicting the prognosis and treatment response. The levels of IL-6, IL-8, IFN-γ, IL-2 are related to the treatment response. The available evidence shows a potential causative role for cytokines in schizophrenia development. There is a substantial need for studies investigating the levels of cytokines before disease development and delineating the therapeutic implications of the disrupted cytokine levels in schizophrenia.

## Introduction

Schizophrenia, a multisystem disorder with a global prevalence of 0.33–0.75%, is one of the top 15 causes of disability (1–4). The underlying etiology of this disease is controversial and not fully understood. Increased dopamine-based activities, together with decreased glutamatergic signaling, are the main suggested etiological hypotheses (5). Abnormalities in the immune system, which are associated with schizophrenia, are one of the other etiological hypotheses.

The immune system is composed of innate and adaptive immune responses. The innate immunity is a rapid-acting antigen-independent response, while the adaptive immunity is an antigen-dependent defense mechanism, with the ability to memorize the antigens. The immune response is mainly mediated by cytokines, which are mostly produced by a critical component of the adaptive immunity, T-lymphocytes. These mediators can be divided into 5 groups; (1) pro-inflammatory cytokines; interleukin (IL)-6, tumor necrosis factor (TNF)-α, IL-1 family, and IL-8, which are involved in initiation and aggravating inflammatory responses (6); (2) T-helper 1 cytokines; IL-2, interferon (IFN)-γ, and IL-12, which create a pro-inflammatory response and function in autoimmune diseases and defense against intracellular parasites; (3) T-helper 2 cytokines; IL- 4, IL-5, and IL-13, which counterbalance the effects of T-helper1 cytokines; (4) T-helper 17 cytokines; Il-17, and IL-23, which are chiefly involve in pro-inflammatory processes and defense against extracellular pathogens; (5) T-regulatory cytokines; IL-10 and transforming growth factor (TGF)-β, which primarily suppress immune responses (7, 8).

The associated immune disorders in schizophrenia have been investigated for more than a century (9–11). These abnormalities include increased activity and density of microglia cells, abnormal profiles of peripheral leukocytes, serum cytokines, and cerebrospinal fluid (CSF) cytokines (12, 13). Based on genome-wide association studies, schizophrenia is also associated with specific major histocompatibility complex (MHC) region genes (14). Furthermore, this disease is significantly linked with enhancers having a strong role in the immune functions, even after excluding the MHC region genes (15). These findings support the clinical and genetic aspects of the connection between schizophrenia and the immune system.

One group of components of the immune system affecting the brain by several mechanisms are cytokines which are either produced outside of the central nervous system (CNS) or within the CNS. Peripheral cytokines, presented in the circulation, can access the CNS and affect it *via* four major ways: (1) binding to specific transporters, (2) stimulating afferent vagal fibers, (3) accessing areas such as circumventricular organs, and (4) passing the damaged blood-brain barrier (BBB) which has an increased permeability (16, 17). Notably, elevated levels of the peripheral markers of BBB damage, such as S100B, indicate BBB damage in schizophrenic patients (18). In addition to the peripheral cytokines, microglia, astrocytes, endothelial cells, and even neurons can produce different cytokines within the CNS (17, 19). Moreover, despite the prevailing view that the brain is an immune-privileged area, several studies have shown that immune responses can be established within CNS by several mechanisms, one of which is transferring the immune cells located within the meninges, which are sources of different immune mediators, into the brain’s parenchyma in a pathologic state. These cells physiologically transfer through the blood-meningeal barrier in order to pass the meninges as it is more permeable than BBB (20, 21).

These cytokines can have various roles in neurodevelopment, neuroendocrine activities, and neurotransmission. Their role in neurodevelopment is mainly through affecting microglia which are the chief cells responsible for this task (11, 16, 17, 19). Cell migration, water balance, body temperature regulation and synthesis and release of neurotransmitters can be influenced by these mediators (16).

A role for cytokines in schizophrenia was proposed almost three decades ago (22, 23). Ever since, an increasing number of studies investigated alterations of cytokine levels in schizophrenic patients, their changes following antipsychotic treatment, and their relationship with clinical manifestations. However, not only are the results of these studies controversial, but they also do not clearly answer whether the changes in the levels of these cytokines can have a causative role in the development of psychotic symptoms. Moreover, diagnostic and therapeutic implications of these alterations are not defined.

This review aims to answer four questions: A) What are the changes in the serum levels of cytokines in schizophrenic patients? B) What is the relationship between the levels of cytokines and the severity of clinical symptoms? C) How antipsychotics affect the baseline levels of cytokines? and D) What is the potential role of cytokines as predictors of treatment response?

Using the answers to these questions, we investigate whether abnormal cytokine levels are the culprit in the pathogenesis of schizophrenia and also provide clinical implications in terms of diagnosis and treatment.

## Alterations in the Levels of Cytokines in Schizophrenic Patients

### Alterations in the Levels of Pro-Inflammatory Cytokines

A considerable number of studies, including meta-analyses, found increased levels of IL-6 in different groups of patients including, first-episode and drug-naive (FEDN) psychosis patients and cases with first-episode psychosis (FEP), majority of whom were using antipsychotics (24–28). Similar findings were observed in chronic patients, including those without any significant inflammation (29), patients in an acute relapse or recovering from it, and stable outpatients (27, 30–32). Recently, Hartwig et al. found increased levels of soluble IL-6 receptors in a clinical two-sample Mendelian randomization study, which can be explained as a compensatory response to the increased levels of IL-6 in schizophrenia (33).

Conversely, some studies found no significant changes in the levels of IL-6 in schizophrenic patients (34, 35).

Multiple studies, including meta-analyses, reported elevated levels of TNF-α, one of the other pro-inflammatory cytokines, in FEDN patients (24, 25), (both adult and pediatric) FEP patients, majority of whom were using antipsychotics (26, 27, 36) and chronically ill patients using antipsychotics, regardless of their status in terms of acute relapse (24, 37). Higher levels of TNF-α have also been reported in chronic patients taking atypical antipsychotics having no major inflammation (29).

On the contrary, Potvin et al. conducted a meta-analysis and found no significant alteration in the levels of TNF-α in *in vivo* and *in vitro* studies (30). Their finding may be explained by the scarcity of studies until 2005. Decreased levels of TNF-α are seen in FEDN patients with a disease duration of under two years (37) and chronic patients with a disease duration of more than five years taking typical and atypical antipsychotics (38, 39).

Regarding the next pro-inflammatory cytokine, IL-8, despite meta-analyses supporting elevated levels of it in FEP patients (27) and chronic patients who are stable or are experiencing an acute relapse or are recovering from one (24, 32), a recent meta-analysis showed no significant alterations in the levels of IL-8 in FEP patients versus HC’s (32). This is concordant with the findings of another study in FEP patients, most of whom were medicated (36). Concordantly, in a study in which less than a quarter of patients were taking benzodiazepine and others were drug-naive, except for obese cases who had increased levels of IL-8, other patients showed no significant alterations in the IL-8 level (40).

IL-1 family is one of the major pro-inflammatory cytokines, one of the members of which is IL-1β. A large number of studies, including several meta-analyses, found elevated levels of IL-1ß in FEDN patients (25) and adult and pediatric FEP patients, majority of whom were taking atypical antipsychotics (26, 28, 36), and chronically ill patients who were stable or were experiencing an acute relapse or were recovering from one (24, 27, 32, 37). Interestingly, it seems that the levels of IL-1ß mRNA do not change in FEP patients (36).

In contrast to a substantial number of studies suggesting an increase in the levels of IL-1ß, the meta-analysis conducted by Potvin et al. in 2008 found no significant alterations in the levels of IL-1ß in *in vivo* and *in vitro* studies (30). No significant elevation has been reported in chronic patients with a disease period of more than six years, either (41). Interestingly, a recent study has found decreased levels of IL-1ß in FEDN patients with a disease period of shorter than 2 years (37).

Other members of the IL-1 family may be elevated in schizophrenic patients as well. The levels of IL-1α and its leukocyte mRNA were found to be higher in FEP patients who were mostly medicated (36). However, other investigators did not find the same pattern in long-term chronically ill patients (41). A recent study has reported increased levels of IL-33, one of the other members of IL-1 family, and its soluble receptor (sST2) in FEDN patients and patients in an acute relapse compared to patients in remission or HC’s (42). Several studies, including a number of meta-analyses, reported increased levels of IL-1 receptor antagonist (IL-1RA) in patients with first-episode psychosis, whether most of the patients used antipsychotics (43) or whether they were drug-naive (40), in chronic patients who were experiencing an acute relapse or had multiple episodes of schizophrenia (24, 27, 32), and in *in vitro* studies (30).

### Alterations in the Levels of T-Helper 1 Cytokines

According to the majority of studies (including meta-analyses), the levels of IL-2, one of the cytokines produced by T-helper 1, do not alter in schizophrenic patients. These studies were performed on FEDN patients, FEP patients (with a history of using antipsychotics in many of them), and chronic patients who were experiencing an acute relapse, were recovering from it or were stable (24, 25, 27, 35, 36).

However, several studies found contradictory results. Some of these studies reported increased levels of IL-2 in FEDN patients with a normal BMI (44), FEP patients, majority of whom were using atypical antipsychotics (28), and chronic patients with stable antipsychotic medication regimens combined with a disease period of more than six years (41). Furthermore, some *in vitro* studies found a substantial decrease in the levels of IL-2 (30). Studies also found a decrease in the mRNA levels of this cytokine in the peripheral blood of chronically ill patients who were using antipsychotics for at least a year (45).

Moreover, several studies (including meta-analyses) found a significant increase in the levels of soluble IL-2 receptor (sIL-2R), levels of which might affect the level of IL-2 by binding with it, in FEDN patients and chronic patients who were stable or were experiencing an acute relapse or were recovering from one (24, 25, 27).

Alterations in IFN-γ, the next T-helper 1 cytokine, levels are very controversial. The correlation between IFN-γ and BMI, previously found in patients with first-episode schizophrenia, may explain this inconsistency (32). Due to this great controversy, the reported changes in the levels of this cytokine in each group of patients are discussed separately.

In FEDN patients, several studies, including a meta-analysis, found no significant alterations in the levels of IFN-γ (40, 46–49) while some other studies, including another meta-analysis, showed increased levels of IFN-γ (24, 50). Conversely, Reale et al. found decreased levels of IFN-γ in FEDN patients (51).

Studies showed elevated levels of this cytokine in adult or pediatric FEP patients, most of whom had a history of using antipsychotic medications (26–28). However, Di Nicola et al. found non-altered levels in adult FEP patients, most of whom were on atypical antipsychotics (36).

Several studies, including some meta-analyses, found elevated levels of IFN-γ in chronic schizophrenic patients who were stable or were experiencing an acute relapse (24, 27, 32). However, no significant disruption was found in patients recovering from an acute relapse (27, 46). Decreased levels of IFN-γ were also reported in patients with acute psychotic symptoms who were drug-naive for at least 6 months (52) and in chronic patients. The details of the medications that were used were not reported in these studies (53, 54).

Investigation of the alterations in the levels of IL-12, one of the other T-helper 2 cytokines, showed that several studies (including meta-analyses) found elevated levels in FEP patients, whether drug-naive or not, and chronic patients who were stable or were experiencing an acute relapse, or were recovering from one (24, 27, 55). In the study by Bedrossian et al., the patients were treated with clozapine for at least one year (55).

In contrast to the studies reporting elevated levels of IL-12, some other studies reported non-altered levels of this cytokine in FEP (28, 43), and in chronic patients who had a history of using antipsychotic medications (32, 45).

### Alterations in the Levels of T-Helper 2 Cytokines

Compared to other T-helper 2 cytokines, there are a larger number of studies performed on the alterations of IL-4. Several studies, including two meta-analyses, found no major differences in the levels of IL-4 between HC’s and FEDN or FEP patients, majority of whom were taking atypical antipsychotics, or chronic patients who had schizophrenia for a long time (including treatment-resistant patients) (25, 27, 30, 36, 48, 56). However, like other cytokines, a small number of studies reported inconsistent results. Decreased levels of IL-4 were reported in chronic patients experiencing an acute relapse, during their treatment after the relapse (supported by a meta-analysis) (27), and in stable chronic patients (41, 57). In addition, elevated levels were reported in FEP pediatric patients taking antipsychotics (26) and in adult chronic patients taking clozapine (58).

Few studies have investigated other T-helper 2 cytokines, including IL-5 and IL-13. Increased levels of IL-5 were found in chronic adult patients with multiple episodes of unsuccessful treatment and FEP pediatric patients who were mostly taking antipsychotics (26, 59). Similarly, the levels of IL-13 have been reported to be elevated in adults with multiple episodes of schizophrenia (32, 59).

### Alterations in the Levels of T-Helper 17 Cytokines

The literature is inconsistent about IL-17 alterations in schizophrenic patients. A recent meta-analysis found no significant changes in the levels of IL-17 in FEDN patients (60). Non-disturbed levels are also reported in chronic patients experiencing an acute relapse (46).

On the contrary, some studies reported increased levels of this cytokine in FEDN (50) and chronic hospitalized patients who were medication free for at least four weeks (61).

However, decreased levels have also been reported in FEDN patients (46) and in chronic patients using different antipsychotics (62).

One of the other main T-helper 17 cytokines is IL-23, which has been reported to be elevated in FEDN and chronic patients in an acute relapse (8, 61, 63).

### Alterations in the Levels of T-Regulatory Cytokines

Goldsmith et al. found reduced levels of IL-10 in FEP patients and chronic patients who were in an acute relapse in their meta-analysis (27). Decreased levels are also reported in FEDN patients (64).

However, this meta-analysis showed that the levels of this cytokine do not change in stable chronic patients (27, 45). Other studies have reported no significant alterations in the levels of IL-10 in comparison with HC in FEDN (44), FEP (36), and chronic patients experiencing an acute relapse (65).

Conversely, many investigators have reported elevated levels of this cytokine in FEDN (49, 66), FEP (26), and chronic patients (41, 57, 67).

Several studies, including meta-analyses, found elevated levels of TGF-β in FEDN, FEP patients (27, 46), and in chronic patients experiencing an acute relapse (47).

However, some studies found other findings. Non-disturbed levels of TGF-β have been detected in FEDN and chronic patients who were medication free for four months before the study (18, 44). Interestingly, some studies found decreased levels of TGF-β in chronic treatment-resistant patients (56).

A summary of alterations in the levels of cytokines in schizophrenia based on the meta-analyses is shown in **Table 1**.

**Table 1:** Summary of alterations in serum levels of cytokines in schizophrenia, based on meta-analyses [18, 20, 21).

Increased levels		Non-altered levels		Increased or non-altered		Increased or decreased or non-altered	
Cytokine	Type	Cytokine	Type	Cytokine	Type	Cytokine	Type
IL-6 TNF-α IL-1β IL-12 TGF-β	PI PI PI TH1 TR	IL-2 IL-4 IL-17	TH1 TH2 TH17	IL-8 IFN-γ	PI TH1	IL-10	TR

IL, interleukin; TNF, tumor necrosis factor; TGF, transforming growth factor; IFN, interferon; PI, pro-inflammatory cytokine; TH1, T-helper 1 cytokine; TH2, T-helper 2 cytokine; TR, T-regulatory cytokine; TH17, T-helper 17 cytokine.

## Relationship Between Cytokine Levels and Severity of Clinical Symptoms

The levels of cytokines seem to be correlated with both the disease duration and symptom severity. Patients with elevated levels of IL-6, IL-8, and IlL-4 tend to have a longer disease duration and longer hospitalizations (31, 65, 68).

Furthermore, higher levels of IL-6, IL-1ß, IL-33, and IL-17 are associated with more severe positive symptoms (28, 42, 50, 69, 70). In chronic patients using a stable dose of antipsychotics, decreased levels of TNF-α are similarly associated with more severe positive symptoms (38, 71), while no correlation has been found in FEDN patients (37).

Exacerbated negative symptoms are seen in patients with elevated levels of IL-6, TNF-α, IL-1ß, IL-8, IFN-γ, IL-4, and TGF-β as well as patients with decreased levels of IL-2 and IL-17 (50, 61, 64, 65, 72–75). Interestingly, the correlation between TNF-α and IL-1ß and negative symptoms is only seen in chronic patients and is not reported in FEDN patients (37). Additionally, in FEDN patients, the levels of IL-10 are negatively correlated with negative symptoms, while in chronic patients, they are positively correlated with these symptoms (54, 76).

Increased levels of IL-6, IL-33, sIL-2R, IL-17, and TGF-β are positively correlated with PANSS (positive and negative syndrome scale) general psychopathology sub-score (42, 50, 61, 65). PANSS is a widely used tool to determine the severity of the psychotic symptoms. It is a clinical interview assessing the severity of positive symptoms, negative symptoms, and general psychopathology in schizophrenic patients *via* 30 items. Higher scores indicate more severe conditions (77). The total PANSS score is positively correlated with the levels of IL-6, sIL-2R, IL-1β, IFN-γ, IL-13, TGF-β1 and IL-17 (61, 65, 78, 79). Interestingly, the levels of IL-6 and IL-17 correlate with the total score in both chronic and FEDN patients, while the levels of IFN-γ correlate with the total score only in FEDN patients (50). Moreover, only in chronic patients, decreased levels of TNF-α are associated with higher general and total sub-scores, and no association has been reported in FEDN patients (37, 38, 71).

Regarding the correlation between IL-8 and severity of symptoms, Dahan and his colleagues did not find any association between the levels of IL-8 and PANSS sub-scores. Instead, they reported that patients with higher levels of IL-8 had higher scores of the Clinical Global Impression (CGI) severity scale (a subjective assessment tool to determine the severity of the mental illness by clinicians) (65, 80). However, the levels of IL-8 are reported to correlate with the PANSS mean score positively (79).

The levels of cytokines may be relevant to behavior disorders as well. Surprisingly, worse cognitive abilities are associated with higher levels of IL-6, IL-1RA, IL-33, and IL-12 or lower levels of TNF-α in chronic patients, and with lower levels of IL-10 in FEDN patients (31, 38, 64, 81–86). Moreover, patients with higher levels of IL-10 had a higher total score of the RBANS (Repeatable Battery for the Assessment of Neuropsychological Status) and a worse performance in the attention domain (83, 87). Furthermore, better performance on the memory and intelligence tests has been reported to be associated with higher levels of IL-2 (72). Aggressive behavior is more common among patients with higher levels of IL-17 and IL-10 (54, 61, 76). In addition, in FEDN schizophrenic patients, depressive behaviors are more prevalent among those who have higher levels of IL-4 and TNF-α (79).

**Table 2** summarizes the relationship between levels of various cytokines and severity of clinical symptoms.

**Table 2:** Relationship between cytokine levels and severity of clinical symptoms.

Negative symptoms		Positive symptoms		Cognitive/intelligence abilities		Total PANSS/RBANS/CGI score		Incidence of depression	Incidence of aggressive behavior
Positive correlation	Negative correlation	Positive correlation	Negative correlation	Positive correlation	Negative correlation	Positive correlation	Negative correlation	Positive correlation	Positive correlation
IL-6 TNF-α IL-8 IL-1β (PI) IFN-γ (TH-1) IL-4(TH2) TGF-β IL-10	IL-2 (TH1) IL-17 IL-10	IL-6 IL-1β IL-33 IL-17	TNF-α	IL-33 TNF-α IL-2 IL-10	IL-6 IL-RA IL-12	IL-6 IL-8 sIL-2R IlL-1β IFN-γ (only in FEDN patients) IL-13 IL-10 TGF-β1 IL-17	TNF-α (only in chronic patients	IL-4	**IL-17** **IL-10**

IL, interleukin; TNF, tumor necrosis factor; TGF, transforming growth factor; IFN, interferon; PANSS, The Positive and Negative Syndrome Scale; RBANS, The Repeatable Battery for the Assessment of Neuropsychological Status; CGI, Clinical Global Impression.

## Effect of Antipsychotics on the Baseline Level of Cytokines

### Altered Pro-Inflammatory Cytokines After Antipsychotic Treatment

Shortly (up to 2 months) after treatment with typical or atypical antipsychotics (such as risperidone), the levels of IL-6 and IL-1β seem to decrease (16, 88, 89). However, in the long term, their levels either rise or do not change compared to the baseline levels (70, 90–92).

The increasing trend of IL-6 and Il-1β can be explained by antipsychotics (especially atypical antipsychotics) side effects such as metabolic syndrome in the long period (93), as increased levels of IL-6, IL-1β, TNF-α, IL-2, IFN-γ, and IL-4 have been reported in patients with metabolic syndrome (94, 95).

### Unaltered Pro-Inflammatory Cytokines After Antipsychotic Treatment

After using typical or atypical antipsychotics for up to two months, the levels of TNF-α and IL-8 do not change (24, 70, 89, 91, 96). The IL-8 levels seem to remain unchanged even after three months of therapy with risperidone and haloperidol (90). However, one study found that taking typical or atypical antipsychotics or a combination of them in FEDN patients for seven months causes a significant decrease in the level of IL-8 while patients’ BMI also increased (97). Furthermore, it has been reported that the levels of TNF-α significantly increase following taking risperidone for more than three months (possibly because of its side effects resulting in induction of metabolic syndrome) (92), or after taking adjunct mood stabilizers with typical or atypical antipsychotics for an average of six weeks of treatment (98). Interestingly, Amisulpride seems to decrease the levels of TNF-α after six weeks of treatment (52).

The level of the anti-inflammatory cytokine, IL-1RA, is reported to decrease following 6 weeks of treatment with olanzapine or risperidone (48) or 8 weeks of antipsychotic therapy adjusted with patients’ clinical status (99). Nonetheless, these studies are in contrast with meta-analyses that found no significant alterations in the levels of this cytokine after an average of eight weeks of treatment (70, 91).

### Altered T-Helper 1 Cytokines After Antipsychotic Treatment

The levels of IL-2 seem to decrease in the first month after antipsychotic therapy (atypical or typical antipsychotics or mixed) (89). Particularly, olanzapine and haloperidol decrease the level of IL-2 significantly (70). However, studies with longer average treatment periods found no significant changes in the level of IL-2 (24, 70, 91).

Moreover, the levels of IL-12 seem to significantly increase following the use of risperidone for 7–8 weeks (24, 91). Similarly, an elevation in the levels of IL-12 has been reported after six weeks of treatment with olanzapine and haloperidol (100). Surprisingly, aripiprazole (a third-generation antipsychotic) seems to decrease IL-12 levels in chronic patients after four weeks of treatment (101). This is in contrast with the results of a meta-analysis of studies with a treatment period of 4–10 weeks using typical or atypical antipsychotics that found no significant changes in the level of IL-12 after medication (70).

### Unaltered T-Helper 1 Cytokine After Antipsychotic Treatment

IFN-γ levels do not significantly change in the first month following antipsychotic treatment (89). However, findings after antipsychotic therapy for an average of approximately 2 months are inconsistent. Two meta-analyses found decreased levels following treatment with typical or atypical or mixed antipsychotics while a meta-analysis by Miller et al., in which more than half of the included studies had non-standardized antipsychotic treatment, suggested the levels of IFN-γ remained constant in this period (16, 70, 91). Olanzapine seems to be the main medication that decreases IFN-γ levels (70). Interestingly, assessing the effect of atypical antipsychotics for 3 months revealed increased levels of IFN-γ, which may be because of their side effects such as metabolic disorder (73).

### T-Helper 2 Cytokines After Antipsychotic Treatment

Several meta-analyses have confirmed no disturbances in the level of IL-4 after an average of two months of antipsychotic treatment (70, 91). In contrast, some studies found that treatment with typical or atypical antipsychotics or a combination of them for 1 month or treatment with risperidone for 10 weeks led to decreased levels of IL-4 (102, 46, 68). Thus, IL-4 might be a trait marker that decreases at first but then increases after a certain time due to the metabolic side effects of antipsychotics, which can result in normal levels in the long term. However, to the best we know, no meta-analysis has evaluated the short-term effects. Furthermore, it has been reported that treatment with atypical antipsychotics for eight weeks causes a significant reduction in the levels of IL-13 (78).

### Unaltered T-Helper 17 Cytokines After Antipsychotic Treatment

IL-17 and IL-23 seem to be a trait marker whose level does not change after one month of treatment with typical or atypical antipsychotics or a combination of them compared to the baseline level (47, 63, 89). Assessing the effect of risperidone for 10 weeks has shown the same result (102). Interestingly, one study found that a 4-week treatment course with risperidone decreased the number of T-h17 cells while it had no significant effects on the IL-17 levels (50).

### Unaltered T-Regulatory Cytokines After Antipsychotic Treatment

The levels of IL-10 do not change following an average of 8 weeks of treatment compared to the baseline levels (70, 91). However, it has been reported that in chronic patients, treatment with aripiprazole, risperidone, or clozapine for 4–6 weeks increased the IL-10 levels (101, 103). The results of treatment with atypical antipsychotics, particularly risperidone, in FEDN patients are different. In these patients, the levels of IL-10 are lower after treatment compared to baseline. Similarly, an average of eight weeks of treatment does not cause a significant alteration in the level of TGF-β (70, 91). However, a meta-analysis by Miller et al., in which more than half of the studies had non-standardized antipsychotic treatment, found decreased levels of TGF-β after 8 weeks of therapy (24). Four weeks of treatment with aripiprazole in chronic patients and 6 weeks of therapy, mostly with atypical antipsychotics in FEDN patients are associated with decreased levels of TGF-β as well (101, 104). However, elevated levels of TGF-β have been reported after four weeks of typical or atypical or mixed antipsychotic treatment in FEDN patients (46).

## The Role of Cytokine Level as Predictors of Treatment Response

There is a growing body of evidence on the clinical implications of cytokines in schizophrenia. Considering the lack of predictor biomarkers of the treatment response in psychosis, one of the suggested applications is using cytokines to predict response to treatment (73). Furthermore, the relationship between cytokine levels and response to treatment can support the hypothesis that cytokines may play a role in schizophrenia pathogenesis.

Increased levels of IL-6 and IFN-γ are associated with treatment resistance (105, 73). Treatment resistance is defined by not achieving the remission criteria proposed by the Schizophrenia Working Group Consensus (106) or Kane’s criteria (107). Patients with higher levels of IL-8 and IL-2 experience less improvement in PANSS compared to other patients after twelve weeks of therapy with risperidone or haloperidol (90). Moreover, even though the level of TGF-β is not related to treatment response, TGF-ß1 polymorphism is reported to be associated with PANSS score improvement after antipsychotic treatment (108).

## Discussion

### Abnormal Cytokine Profiles in Schizophrenic Patients

The results showed that schizophrenic patients had an inflammatory cytokine profile and imbalanced T-helper 1, T-helper 2, and regulatory cytokines. The severity of symptoms and abnormal behaviors in addition to antipsychotic therapies may affect abnormal cytokine levels in these patients. However, there were significant inconsistencies regarding the cytokine profile in the reviewed studies in schizophrenic patients. Six reasons may explain these controversies: 1) Diverse Patients’ characteristics; 2) Different sampling methods; 3) Heterogeneous patient populations; 4) The difference between cytokine profile of plasma, serum or whole blood; 5) Different specifications of assay kits; 6) Small sample size.

1. Diverse Patients’ characteristics: Several studies did not evaluate factors such as the BMI, diet, diurnal rhythm, smoking habits, psychological stress, and lifestyle in their patients, which may affect the cytokines profile (109–112). However, elevated levels of IL-6 are reported in a study excluding patients with a body mass index (BMI) > 25, which gave rise to the conclusion that the changes of IL-6 did not seem to be related to obesity (44).
2. Different sampling methods: The method and duration of storage and the used anticoagulant may affect the measurement of levels of cytokines. Moreover, as the levels of cytokines are influenced by the circadian pattern, the time of sampling is of great importance. Mornings are suggested to be the best time to take samples (113).
3. Heterogeneous patient populations: In some studies, FEDN patients were not separated from patients with a history of antipsychotic treatment. Not only do antipsychotics affect cytokine levels, but they can also cause weight gain, which is considered a low-grade inflammation (93). Thus, studies in a FEDN population are more reliable.
4. The difference between cytokine profile of plasma, serum or whole blood: The cytokine profile of each of these can be different from the other ones as the coagulation process may trigger release of some inflammatory cytokines (113).
5. Different specifications of assay kits: The quality of antibody used in ELISA kits, kit manufacture, and the operator’s skills may affect the measurement (113). For example, Hope et al. indicated that different assay kits could affect the measurement of serum levels of different cytokines. In their study, the IL-6 level was higher than the detection limit of the kit in more than half of the samples (82).
6. Small sample size: A considerable number of the studies had small sample sizes (less than 30 participants in each of their groups) that limited their statistical power (54, 114).

### Abnormal Cytokine Profile: Culprit, Consequence, or Simple Association?

The question is whether abnormalities in cytokine levels have a causative role in schizophrenia or are merely associated with the disease? We used the Bradford Hill criteria (115, 116) to answer this question. These criteria assess nine factors, including the strength of association, consistency, specificity, temporality, biological gradient, plausibility, coherence, analogy, and experimental effect to differentiate causality from association. As for the strength of association, the levels of several cytokines (specifically pro-inflammatory cytokines) are found to be significantly disturbed in schizophrenic patients. The second criterion, consistency, is not met in most of the cytokine level alterations. These changes are not specific either and can cause a wide variety of diseases. However, in the modern context, specificity is a less important factor in determining or refusing causality (116). Temporality, as the next criterion, was assessed by only a few studies. In two studies that measured the levels of cytokines before schizophrenia presentation, individuals who developed the disease in the following years had higher baseline levels of IL-6 compared to others (117, 118). This association is strengthened by the study of Khandaker et al. in 2018, finding a strong association between a genetic variant of IL-6 receptor relating to the levels of IL-6 and CRP and development of schizophrenia (119). Moreover, individuals with higher levels of inflammatory markers, including ESR (erythrocyte sedimentation rate) and CRP (C-reactive protein), were more likely to develop schizophrenia (120, 121). The severity of the psychotic symptoms correlated with abnormalities in the levels of cytokines, particularly pro-inflammatory cytokines. So, the biological gradient criterion was met. Regarding plausibility and coherence, there are three suggested major ways by which pro-inflammatory cytokines can contribute to schizophrenia development. First, they can increase kynurenic acid (a metabolite of tryptophan) formation. This metabolite function as an N-Methyl-D-aspartate (NMDA) receptor antagonist, which based on the glutamate hypothesis of schizophrenia (122), might play a causative role in schizophrenia together with decreased glutamatergic signaling. Second, these cytokines increase oxidative stress leading to increased neurodegeneration, which can be seen in schizophrenia. Third, pro-inflammatory cytokines may disturb neurodevelopment (particularly when there is a prenatal inflammation), increasing the risk of psychosis (12, 123). Furthermore, the interplay of cytokines and neurotransmitters may be one of the mechanisms by which cytokines can play a causative role in schizophrenia. Inflammatory cytokines can affect synthesis of monoamine neurotransmitters, increase reuptake of dopamine, serotonin, and norepinephrine, and influence on the release of neurotransmitters (16). Increased levels of soluble IL-6 receptors can be explained as a compensatory response to increased levels of IL-6 (33), and elevated levels of TNF-α mRNA suggest systemic blood immune cells as the main source of higher levels of TNF-α (36). These two findings provide more experimental evidence for this causation. In terms of analogy, disrupted cytokine levels can be seen in depression and bipolar disorder (114). There is considerable evidence supporting the causal role of cytokines in depression (124). Finally, decreasing inflammation using adjuvant anti-inflammatory agents is reported to improve the symptoms in schizophrenic patients. Consequently, the experimental effect criterion was met as well (125, 126).

## Clinical Implications

### Early Diagnosis

The association between cytokine alterations and schizophrenia may have potential clinical implications. Currently, the diagnosis of schizophrenia is based on clinical symptoms, and there is no standard diagnostic biomarker that allows early recognition of this disease, while cytokines such as IL-6, TNF-α, IL-1ß, and IL-RA can be used as potential biomarkers for early detection of at least a subgroup of schizophrenic patients (118, 127, 128). Furthermore, IL-6, sIL-2r, TNF-α, IL-1RA, and IL-4t can also be useful in detecting the acute-relapse phase of disease (27, 46).

### Novel Therapeutic Horizons

Targeting inflammatory pathways may lead to new treatment options in schizophrenia. A growing body of evidence supports the effect of immune modulators on ameliorating the symptoms of schizophrenia. (12, 129). A recent meta-analysis showed that a variety of medications can reduce the severity of symptoms of schizophrenia. These medications are aspirin (by reducing inflammation by modifying cyclooxygenase-2 enzyme), estrogens (through immunomodulatory effects), minocycline (by inhibiting microglia), and N-acetylcysteine (as an anti-inflammatory agent) (130).

### Prediction of Prognosis

Prediction of response to treatment is another application of cytokine levels. Patients with increased levels of IL-6, IL-8, IFN-γ, and soluble TNF-α receptor 1 and decreased levels of IL-2 exhibit less improvement after standard antipsychotic therapy (73, 90, 131). Moreover, patients with higher levels of CRP, an inflammatory marker, tend to have lower quality of life (132).

### Patient Stratification for Choosing the Best Therapeutic Protocol

It is possible that patients who have higher levels of pro-inflammatory cytokines benefit more from adding specific drugs to the standard therapeutic regimen. Stratifying patients on this basis can potentially help the physicians choose the best treatment option. A randomized clinical trial study in treatment-resistant depressive patients supports this hypothesis. Depression is also associated with a disrupted cytokine profile. This study showed that in a subgroup of patients who had higher baseline inflammation, the use of infliximab—a TNF-α antagonist—led to better results after the treatment. However, infliximab had no significant effects on another subgroup of patients (133).

### Future Studies: Causation, Diagnosis, Prognostic, and Therapeutic Applications

Although current studies provide a wealth of information on the cytokines profile in schizophrenic patients, several major shortcomings cannot be overlooked. There is a substantial need for longitudinal studies investigating the levels of cytokines before the development of clinical manifestations of schizophrenia in individuals with a strong positive family history of schizophrenia. Moreover, the potential confounding factors such as age, sex, smoking, obesity, and individuals’ diet should be rigorously controlled in the future studies (134).

More studies also need to be performed on the diagnostic and the prognostic applications of measurement of levels of cytokines. The relationship between severity of symptoms and levels of cytokines in FEDN patients can be one of the examples of the diagnostic applications. Identifying treatment-resistant patients based on their cytokines profile can be noted as the prognostic applications, on which more studies are needed. Last but not the least, future studies should be based on defined categories of schizophrenic patients.

Lastly, there is a significant shortcoming in the studies that investigate the therapeutic applications. As an example, selection of patients for adjuvant therapy with medications targeting immune modulatory pathways can be guided by inflammatory cytokine levels (135). Moreover, the role of adjuvant monoclonal antibody immunotherapy, which in contrast to NSAIDs only targets immune pathways, needs to be more investigated (134).

## Limitations

This was a narrative review. Therefore, all the limitations of narrative reviews may apply to this study (136).

## Conclusion

Schizophrenia is associated with several abnormalities in the levels of cytokines, which are the mediators of the immune system. A deeper understanding of this association can be useful in clinical practice in terms of early diagnosis and treatment.

## Author Contributions

SM developed the concept and design, collected the data, drafted the article, critically revised the manuscript for important intellectual content, and approved the final version. AZ-S critically revised the manuscript for important intellectual content and approved the final version. NR supervised the project, critically revised the manuscript for important intellectual content, and approved the final version.

## Conflict of Interest

The authors declare that the research was conducted in the absence of any commercial or financial relationships that could be construed as a potential conflict of interest.
